# Relational learning cues, music receptivity, and performance self-efficacy among piano masterclass participants: A PLS-SEM and fsQCA study

**DOI:** 10.1371/journal.pone.0361767

**Published:** 2026-11-03

**Authors:** Yixuan Zhao, Xiaohui Zhu, Yan Su

**Affiliations:** 1 Faculty of Music, Moscow Pedagogical State University (MPGU), Moscow, Russia; 2 Moscow Conservatory, Moscow, Russia; 3 Institute of Arts, Moscow Pedagogical State University (MPGU), Moscow, Russia; 4 Repin Academy of Arts, Saint Petersburg, Russia; 5 Qilu Normal University, Jinan, China; East China Normal University, CHINA

## Abstract

**Background:**

Performance self-efficacy is important for musical learning, yet research on its correlates in masterclass settings has mainly emphasized instruction and individual practice. Drawing on social cognitive theory and relationship-interaction-based brand equity, this study examined whether five relational cues in piano masterclasses—brand personality, reciprocity, courtesy, relationship status, and relationship energy—were associated with participants’ performance self-efficacy and with affective and attentional music receptivity.

**Methods:**

A cross-sectional online survey was completed by 382 adult and adolescent participants with piano-masterclass experience in mainland China. PLS-SEM tested the hypothesized structural paths. Model evaluation included HTMT, full-collinearity VIFs, SRMR, and ten specific indirect associations estimated with 5,000 two-tailed bias-corrected and accelerated (BCa) bootstrap samples. fsQCA results were examined under stricter frequency, consistency, PRI, and calibration specifications.

**Results:**

Reciprocity, courtesy, relationship status, and relationship energy were positively associated with performance self-efficacy, whereas brand personality had no significant direct association. Affective receptivity was positively associated with reciprocity, courtesy, status, and energy; attentional receptivity was positively associated with personality, reciprocity, and status. Both receptivity dimensions were positively associated with self-efficacy. Seven of ten specific indirect associations had BCa intervals excluding zero. The baseline fsQCA solution contained seven configurations; stricter robustness specifications yielded fewer configurations, although recurring combinations of relational cues and music receptivity remained.

**Conclusion:**

Performance self-efficacy in piano masterclasses was associated not only with instructional content but also with relational and experiential cues. Affective and attentional music receptivity represented distinct intervening statistical pathways. Because the survey was cross-sectional, retrospective, and based on convenience recruitment, the results do not establish causality or temporal mediation and should be generalized cautiously beyond this predominantly adult, highly educated, music-trained sample.

## 1 Introduction

Performance self-efficacy refers to individuals’ beliefs in their capability to organize and execute the courses of action required to attain designated performance outcomes [1]. In music, it denotes a learner’s judgment of the capability to perform successfully. Such beliefs are related to cognitive, affective, and volitional processes, including effort, persistence, practice strategies, responses to difficulty, and performance outcomes [2,3]. Recent work in piano education likewise links self-efficacy with reflective learning and practice behavior [4]. Identifying the instructional and social correlates of performance self-efficacy is therefore important.

Existing music-education research has emphasized mastery experience, guided practice, modeling, feedback, self-assessment, social support, and goal setting. Individual lessons and group performance can provide opportunities to accumulate successful experiences; immediate, specific feedback and guided reflection may also help learners interpret their progress. Social cognitive theory identifies mastery experience, vicarious experience, verbal persuasion, and physiological or affective states as principal sources of efficacy information [1]. Music-specific studies similarly show that efficacy beliefs are related to prior achievement, performance experiences, and perceived support [3,5]. Goal-setting theory further supports the motivational value of specific and attainable goals [6].

Piano masterclasses are distinctive learning settings rather than ordinary individual lessons. They bring a student performer, an expert musician-teacher, and an audience into a public episode of performance, diagnosis, modelling, and feedback. Hanken’s research shows why this arrangement matters: it gives performers access to a fresh professional perspective and concert-like practice, exposes them to standards embodied by an established musician, and allows audience members to learn through observation, comparison, peer assessment, and reflection [7–10]. These opportunities are not automatic. The public format can also heighten vulnerability, and the teacher must address the performer while making the episode intelligible to observers. A masterclass is therefore both an instructional encounter and a relationally charged public learning environment.

This study does not treat piano education as a consumer transaction. It uses relationship-interaction-based brand equity as an analytical vocabulary for the reputational, relational, and symbolic meanings that participants attach to a focal pianist/master teacher. Respondents were instructed to recall one specific piano masterclass and answer with reference to that pianist/master teacher; “brand” therefore refers operationally to the focal teacher, not to a commercial product. Some respondents may nevertheless have incorporated the masterclass series or organizing institution into their judgments, and this possible referent spillover is acknowledged as a limitation. The five adapted dimensions were brand personality, reciprocity, courtesy, relationship status, and relationship energy [11].

The conceptual move from brand equity to relational learning cues requires clear boundaries. Personality denotes the perceived professional and expressive character of the focal pianist/master teacher. Reciprocity denotes whether participants perceive worthwhile educational returns for their effort. Courtesy concerns respectful treatment, status concerns recognition and trust within the relationship, and energy concerns enthusiasm and willingness to maintain engagement [8,10] helps sharpen these distinctions. The master teacher’s artistic standing can legitimize feedback and embody professional standards, but prestige may also provide status or career symbolism without guaranteeing educational value. By contrast, a safe and respectful climate, individualized recognition, useful advice, and visible responsiveness can enable performers to accept challenge and observers to appropriate the episode for their own development. The present constructs therefore separate teacher persona and status from the reciprocity, courtesy, and energy experienced in the interaction [8,10,11]. The pedagogical value of a masterclass cannot be inferred from prestige or market visibility alone.

The masterclass format also makes receptivity theoretically relevant. A participant may be emotionally moved without sustaining careful attention to demonstrations, or may attend closely without reporting strong emotional resonance. George and Ilavarasu [12] treat these as distinguishable dimensions of music receptivity. Social cognitive theory provides a reason to connect both dimensions with efficacy judgments: affective states can be interpreted as information about capability, while attention is necessary for observational learning and for encoding modeled actions [1].

The study addresses three gaps. First, music self-efficacy research has provided strong accounts of individual practice, prior achievement, feedback, and sources of efficacy information, but has paid less attention to how a public learning encounter is interpreted relationally. Second, Hanken’s program of masterclass research identifies fresh expert perspectives, concert-like performance practice, professional socialization, observational learning, peer comparison, and the need for a constructive climate [7–10]. It does not, however, quantify how participants’ perceptions of teacher persona, reciprocity, courtesy, status, and relational energy are differently associated with affective and attentional receptivity or performance self-efficacy. Third, an average-association model may miss equifinality: different combinations of recognition, reciprocity, courtesy, energy, personality, emotion, and attention may correspond with the same high-confidence outcome. The study therefore uses PLS-SEM to estimate direct and specific indirect statistical associations and fsQCA to examine configurations associated with high performance self-efficacy.

## 2 Research hypotheses

### 2.1 Interactive brand equity of piano masterclasses and students’ performance self-efficacy

Brand personality represents human-like traits and values attributed to the focal pianist/master teacher [13]. A distinctive, positive professional persona may provide a salient model and an aspirational identity cue. Social cognitive theory proposes that people can form efficacy beliefs partly through observing credible models and interpreting their own similarity to, and attainable progression toward, those models [1]. In a piano masterclass, perceived rigor, creativity, or vitality may therefore be positively related to a participant’s confidence. Hypothesis 1: Brand personality is positively associated with performance self-efficacy.

Brand reciprocity refers to the perception that the focal teacher or masterclass provides fair and meaningful returns for participants’ effort, time, and investment. Personalized guidance, useful feedback, and access to learning resources can be interpreted as recognition and social support. Within social cognitive theory, supportive evaluation and verbal persuasion can contribute to efficacy beliefs [1], while relationship-marketing research identifies reciprocity and gratitude as important features of continuing exchange relationships [14]. Hypothesis 2: Brand reciprocity is positively associated with performance self-efficacy.

Brand courtesy refers to perceived friendliness, care, understanding, and respect. In a masterclass, it may be expressed through respectful communication, patient guidance, constructive feedback, and sensitivity to individual needs. Such treatment can reduce interpersonal threat and provide verbal and social information from which learners interpret their capabilities. Hypothesis 3: Brand courtesy is positively associated with performance self-efficacy.

Relationship status refers to perceived recognition, trust, familiarity, and standing in the relationship with the focal pianist/master teacher. Feeling recognized by the teacher or masterclass community may be associated with belonging and competence, both of which can support efficacy judgments [1]. Hypothesis 4: Relationship status is positively associated with performance self-efficacy.

Relationship energy captures vitality, enthusiasm, and willingness to continue engaging with the focal teacher or masterclass. Positive affect is one source from which people interpret efficacy, and an energizing interaction may be associated with persistence and motivated engagement [1]. Hypothesis 5: Relationship energy is positively associated with performance self-efficacy.

### 2.2 Interactive brand equity of piano masterclasses and music receptivity

Music receptivity was examined as a possible intervening statistical pathway between the five relational cues and performance self-efficacy. Affective music receptivity concerns feelings and emotions evoked while listening to or watching the focal pianist; attentional music receptivity concerns sustained focus on the performance and instructional material despite possible distractions [12].

A distinctive and positive professional persona may make a pianist’s performance more emotionally salient. Brand-personality research suggests that human-like brand meanings can support emotional attachment [13,15]. In a masterclass, perceived sincerity, competence, excitement, or sophistication may similarly correspond with stronger emotional resonance. Hypothesis 6: Brand personality is positively associated with affective music receptivity.

Brand personality may also be related to attention allocation. In a setting with many competing cues, a distinctive, coherent, and engaging professional persona can make the focal teacher and musical content more salient. Social cognitive theory assigns attention a central role in observational learning [1]. Hypothesis 7: Brand personality is positively associated with attentional music receptivity.

Reciprocity describes a mutually beneficial exchange in which participants perceive worthwhile educational returns for their effort. In relationship research, perceived returns and gratitude are associated with positive responses and continuing engagement [14]. In a masterclass, perceived reciprocity may likewise correspond with belonging, satisfaction, and stronger emotional engagement. Hypothesis 8: Brand reciprocity is positively associated with affective music receptivity.

Reciprocity cues may also correspond with attention. When participants perceive that the focal teacher provides worthwhile guidance and responds constructively to their investment, they may allocate more cognitive resources to the learning encounter. Hypothesis 9: Brand reciprocity is positively associated with attentional music receptivity.

Courtesy may correspond with affective receptivity because respectful interaction can reduce interpersonal threat and allow participants to engage more openly with the musical experience. This proposed contextual association is tested directly rather than treated as an established educational effect. Hypothesis 10: Brand courtesy is positively associated with affective music receptivity.

Thoughtful and attentive courtesy may reduce interpersonal distraction and help participants remain engaged with the current learning activity. Hypothesis 11: Brand courtesy is positively associated with attentional music receptivity.

Perceived relationship status may correspond with feelings of self-worth, recognition, and belonging, and therefore with affective engagement. Hypothesis 12: Relationship status is positively associated with affective music receptivity. Status may also correspond with attention when recognition within the learning encounter encourages goal-directed engagement and persistence [6]. Hypothesis 13: Relationship status is positively associated with attentional music receptivity.

Relationship energy captures enthusiasm and willingness to sustain engagement. A lively and supportive interaction may correspond with more positive emotions during the musical experience and with greater allocation of attention to the learning encounter. Hypothesis 14: Relationship energy is positively associated with affective music receptivity. Hypothesis 15: Relationship energy is positively associated with attentional music receptivity.

### 2.3 Music receptivity and performance self-efficacy

According to social cognitive theory, people use physiological and affective states when judging capability [1]. Enjoyment, excitement, or emotional resonance during music learning may therefore correspond with more favorable interpretations of competence. Hypothesis 16: Affective music receptivity is positively associated with performance self-efficacy.

Attention is required for observational learning and for using demonstrations and feedback effectively [1,16]. Sustained attention may therefore correspond with stronger perceived mastery of the repertoire and greater confidence. Hypothesis 17: Attentional music receptivity is positively associated with performance self-efficacy. The implied indirect paths are products of contemporaneous associations and do not demonstrate temporal mediation.

H16 and H17 connect the two receptivity dimensions with performance self-efficacy. The implied indirect paths are theoretically meaningful, but the one-wave design places a strict limit on their interpretation. An indirect coefficient is the product of contemporaneous associations; it does not demonstrate that a relational cue occurred first, changed receptivity second, and changed efficacy third. The hypotheses are therefore evaluated as an associational system, and the specific indirect estimates are reported with BCa intervals without claiming temporal mediation.

## 3. Research design

### 3.1 Research methods

This study used complementary PLS-SEM and fuzzy-set qualitative comparative analysis (fsQCA) [17]. PLS-SEM estimated average net associations among the constructs and tested the 17 prespecified structural paths. fsQCA then examined whether different combinations of the seven antecedent conditions were consistently associated with high performance self-efficacy. The two methods answer different questions: PLS-SEM summarizes symmetric net associations, whereas fsQCA evaluates set-theoretic sufficiency and equifinality.

fsQCA calibrates variables as degrees of membership in sets and evaluates asymmetric configurations. Consistency indicates the extent to which cases with a condition or configuration also belong to the outcome set; coverage indicates empirical relevance. Parsimonious and intermediate solutions can be compared to distinguish conditions appearing in both solution types from those appearing only in the intermediate solution [18]. Set-theoretic sufficiency is not equivalent to experimental causality.

PLS-SEM was selected because the model contains several simultaneous reflective constructs and emphasizes prediction and explained variance. Measurement evaluation preceded structural interpretation. Loadings, Cronbach’s alpha, rho_A, composite reliability, and AVE were used to evaluate indicator reliability and convergent validity; Fornell–Larcker and HTMT were used to evaluate discriminant validity. Structural reporting includes path coefficients, confidence intervals, f^2^, R^2^, Q^2^, and predictive comparisons. HTMT ratios, full-collinearity VIFs, and SRMR were calculated from the anonymized item-level data; the procedures and complete numerical outputs are provided in S3 File.

Sample adequacy was considered in relation to the smallest path likely to be substantively relevant, not only the ten-times heuristic. Using the inverse-square-root approximation with alpha = .05, power = .80, and n = 382, the analysis can detect a standardized path of approximately 0.14. This value is close to the smallest supported structural paths in the estimated model. The indirect-association analysis used BCa limits, including jackknife-derived acceleration, and a fixed seed to make the results reproducible.

The fsQCA analysis began with calibration because configurational results depend on how raw scores are translated into set membership. Empirical percentile anchors were used rather than mechanically treating Likert endpoints as populated qualitative anchors. Truth-table rows were evaluated using case frequency, raw consistency, and PRI consistency. Because the number of configurations identified changed under stricter specifications, robustness is interpreted as evidence about the stability of broader patterns rather than proof that every baseline recipe is invariant.

### 3.2 Questionnaire design and data collection

#### 3.2.1 Questionnaire design.

The questionnaire measured eight reflective constructs using 26 substantive items and a five-point Likert-type response scale (1 = strongly disagree, 2 = disagree, 3 = neither agree nor disagree, 4 = agree, and 5 = strongly agree). Seventeen relationship-interaction items were adapted from Zhong and Zhang [11] by replacing the commercial-brand referent with the focal pianist/master teacher and masterclass context. Six affective and attentional music-receptivity items were adapted from George and Ilavarasu [12]. Three performance self-efficacy items were contextualized from the GSE-6 framework of Romppel et al. [19] for piano-masterclass performance. Börekci et al. [20] and Ou and Qin [21] informed the music-performance context but were not the source of the three administered items. The complete administered Chinese items and faithful English translations, together with construct sources and adaptation notes, are supplied in S2 File.

Because the measures came from both Chinese- and English-language sources and all required contextual adaptation, questionnaire development proceeded in stages. Zhong and Zhang’s [11] Chinese items were reworded for the focal pianist/master teacher and the masterclass setting. The English-language receptivity and self-efficacy items underwent forward and back translation: a bilingual researcher produced the initial Chinese version, another bilingual researcher independently back-translated it into English, and discrepancies were resolved through comparison with the source wording. Reviewers familiar with music education and questionnaire design then examined semantic equivalence, clarity, and contextual relevance. The resulting questionnaire was pilot-tested with people who had piano-masterclass experience to assess comprehension, completion flow, response-option use, and display on Wenjuanxing. Ambiguous expressions were refined before formal data collection, and pilot responses were not included in the final analytic sample.

Immediately before the substantive items, respondents received a focal-referent instruction. Its English rendering was: “Please recall one specific piano masterclass that you have attended or in which you performed. Choose a session you remember clearly. In the following statements, ‘this pianist/master teacher’ refers only to the pianist/master teacher in that selected session, and ‘this masterclass’ refers to the same session. Please answer every statement on the basis of that one experience, rather than your general impression of pianists, a masterclass series, or the organizing institution. There are no right or wrong answers. Select the response that best describes your experience, from 1 (strongly disagree) to 5 (strongly agree).” This instruction established a common referent for all eight constructs and reduced ambiguity between the teacher, the event, and the host institution.

#### 3.2.2 Data collection.

The target population consisted of individuals in mainland China who had previously participated or were currently participating in piano masterclasses. Data collection took place in July–August 2025 via the online platform Wenjuanxing (WJX/Sojump). Participation was entirely voluntary, with a small gift provided as an incentive. The final questionnaire was administered in Chinese; English translations are provided in S2 File for transparency. Before completing the questionnaire, participants were informed of the study purpose, the voluntary nature of participation, and their right to withdraw at any time.

The study was approved by the Ethics Review Committee of Qilu Normal University on January 23, 2025 (Ethical Approval No. xsllsc2025−028). Before the questionnaire opened, participants viewed information about the study purpose, voluntary participation, withdrawal rights, and data use. Adults provided informed electronic written consent by clicking “Agree”; only respondents who clicked “Agree” could proceed. For participants younger than 18 years, a parent or legal guardian reviewed the study information and provided informed electronic written consent by clicking “Agree” before the participant could proceed. Qilu Normal University provided local ethics oversight because recruitment and data collection occurred in China; the authors’ Moscow affiliations represent their academic appointments rather than the data-collection site.

After consent and eligibility screening, respondents completed the 26 substantive items with reference to the selected masterclass and then answered the demographic questions. To reduce common-method concerns, the questionnaire used neutral wording, emphasized anonymity, maintained a consistent focal referent, and placed demographics at the end. Duplicate records with identical key fields and IP addresses were removed; IP addresses were then deleted. No direct identifiers were retained.

PLS-SEM estimation used reflective Mode A measurement and 5,000 bootstrap resamples with two-tailed tests and 95% bias-corrected confidence intervals. Reliability and convergent validity were assessed using loadings, Cronbach’s alpha, rho_A, composite reliability, and AVE; discriminant validity was evaluated using Fornell–Larcker and HTMT. SRMR was used as a model-fit diagnostic. The inverse-square-root method indicated that n = 382 was sufficient to detect a standardized path of approximately 0.14 at alpha = .05 and 80% power. Complete HTMT, VIF, SRMR, and indirect-association results are reported in S3 File.

#### 3.2.3 Sample profile.

The final sample comprised 382 respondents: 198 men (51.8%) and 184 women (48.2%). Ages were under 15 (n = 2, 0.5%), 15–20 (n = 22, 5.8%), 21–25 (n = 111, 29.1%), 26–30 (n = 190, 49.7%), 31–40 (n = 49, 12.8%), 41–50 (n = 3, 0.8%), and 51–60 (n = 5, 1.3%). Educational attainment was junior middle school (n = 2, 0.5%), high school (n = 14, 3.7%), bachelor’s degree (n = 79, 20.7%), master’s degree (n = 170, 44.5%), and doctoral study or degree (n = 117, 30.6%). Most participants had a music-related educational background (n = 364, 95.3%). The sample was therefore predominantly adult, highly educated, and music-trained. The near-even gender distribution may reflect the recruitment channels and should not be assumed representative of all Chinese piano learners. Income source and income level were not available in the analytic dataset and were therefore not analyzed.

## 4. Data analysis and results

### 4.1 Reliability and validity assessment

Reliability and validity were assessed using SPSS 23.0 and the SmartPLS output. All item loadings exceeded 0.80. Composite reliability ranged from 0.883 to 0.917, and AVE ranged from 0.687 to 0.771, supporting convergent validity. The Fornell–Larcker results in Table 1 showed that each square root of AVE exceeded the construct’s correlations with the other constructs.

**Table 1:** Fornell–Larcker correlation matrix and discriminant-validity assessment.

	Attentional music receptivity	Brand Personality	Courtesy	Affective music receptivity	Energy	Performance Self-Efficacy	Reciprocity	Status
Attentional music receptivity	0.846							
Courtesy	0.121	0.219	0.856					
Affective music receptivity	0.242	0.217	0.306	0.862				
Energy	0.236	0.210	0.108	0.397	0.866			
Performance Self-Efficacy	0.333	0.161	0.358	0.522	0.376	0.878		
Reciprocity	0.387	0.209	0.227	0.421	0.245	0.414	0.871	
Status	0.366	0.161	0.142	0.419	0.218	0.375	0.219	0.870

Calculations based on the anonymized item-level data yielded HTMT ratios from 0.129 to 0.621, full-collinearity VIFs from 1.222 to 1.735, and SRMR = 0.047. The VIF result provides no indication of pathological full collinearity but does not prove that common-method bias is absent [22]. Complete diagnostics are reported in S3 File.

The measurement results should be read in light of the contextual adaptation. Reliability coefficients and convergent-validity statistics indicate that items within each construct were strongly related, while the Fornell–Larcker and HTMT results indicate adequate empirical separation among constructs. These statistics do not by themselves establish content equivalence between the original brand setting and the masterclass setting. S2 File therefore reports every administered Chinese item alongside a faithful English translation and identifies the source and adaptation for each construct.

Common-method bias is a concern because predictors and outcomes were self-reported in the same survey. Procedural steps reduced some risks but cannot eliminate shared response tendencies. The full-collinearity VIF diagnostic is reported as one statistical check, not as a definitive test. All VIFs were below 3.3, and the HTMT and Fornell–Larcker patterns did not indicate construct collapse. Nevertheless, possible common-rater effects remain a limitation and are another reason that structural paths are interpreted as associations.Table 2 presents the measurement-model assessment, including factor loadings, Cronbach’s alpha, rho_A, AVE, and composite reliability.

**Table 2:** Measurement model assessment.

Measurement Items	Factor Loadings	Alpha	rho_A	AVE	CR
Attentional music receptivity		0.801	0.802	0.715	0.883
Attentional music receptivity1	0.846				
Attentional music receptivity2	0.829				
Attentional music receptivity3	0.861				
Brand personality		0.886	0.888	0.687	0.917
Brand personality1	0.830				
Brand personality2	0.822				
Brand personality3	0.840				
Brand personality4	0.810				
Brand personality5	0.842				
Courtesy		0.818	0.827	0.732	0.891
Courtesy1	0.842				
Courtesy2	0.863				
Courtesy3	0.861				
Affective music receptivity		0.827	0.828	0.743	0.897
Affective music receptivity1	0.861				
Affective music receptivity2	0.864				
Affective music receptivity3	0.861				
Energy		0.833	0.840	0.750	0.900
Energy1	0.885				
Energy2	0.867				
Energy3	0.846				
Performance Self-Efficacy		0.851	0.852	0.771	0.910
Performance Self-Efficacy1	0.892				
Performance Self-Efficacy2	0.863				
Performance Self-Efficacy3	0.879				
Reciprocity		0.841	0.841	0.759	0.904
Reciprocity1	0.867				
Reciprocity2	0.870				
Reciprocity3	0.876				
Status		0.839	0.840	0.757	0.903
Status1	0.874				
Status2	0.867				
Status3	0.870				

### 4.2 PLS-SEM analysis

PLS-SEM was used to estimate the structural model. The estimated path coefficients and bias-corrected confidence intervals are reported in Table 3. For attentional music receptivity, brand personality (β = 0.308, 95% CI [0.223, 0.394]), reciprocity (β = 0.265, [0.170, 0.357]), and status (β = 0.253, [0.161, 0.343]) had intervals excluding zero; courtesy (β = −0.049, [−0.139, 0.041]) and energy (β = 0.057, [−0.027, 0.142]) did not. For affective music receptivity, courtesy (β = 0.176, [0.095, 0.259]), energy (β = 0.249, [0.171, 0.331]), reciprocity (β = 0.252, [0.168, 0.336]), and status (β = 0.280, [0.188, 0.366]) had intervals excluding zero; personality (β = 0.028, [−0.065, 0.120]) did not. For performance self-efficacy, courtesy, energy, reciprocity, status, affective receptivity, and attentional receptivity had positive intervals excluding zero, while personality did not. The relational cues therefore showed differentiated associations with the two receptivity dimensions and self-efficacy.

**Table 3:** Path coefficients of the structural model.

Hypotheses	Path Coefficient	Bias corrected Confidence Interval	Decisions	
H1	Brand Personality → Performance Self-Efficacy	−0.083	[-0.170, 0.004]	Not supported
H2	Brand Reciprocity → Performance Self-Efficacy	0.155	[0.066, 0.246]	Supported
H3	Brand Courtesy → Performance Self-Efficacy	0.210	[0.129, 0.292]	Supported
H4	Relationship Status → Performance Self-Efficacy	0.133	[0.043, 0.219]	Supported
H5	Relationship Energy → Performance Self-Efficacy	0.172	[0.092, 0.251]	Supported
H6	Brand Personality → Affective music receptivity	0.028	[-0.065, 0.120]	Not supported
H7	Brand Personality → Attentional music receptivity	0.308	[0.223, 0.394]	Supported
H8	Brand Reciprocity → Affective music receptivity	0.252	[0.168, 0.336]	Supported
H9	Brand Reciprocity → Attentional music receptivity	0.265	[0.170, 0.357]	Supported
H10	Brand Courtesy → Affective music receptivity	0.176	[0.095, 0.259]	Supported
H11	Brand Courtesy → Attentional music receptivity	−0.049	[-0.139, 0.041]	Not supported
H12	Relationship Status → Affective music receptivity	0.280	[0.188, 0.366]	Supported
H13	Relationship Status → Attentional music receptivity	0.253	[0.161, 0.343]	Supported
H14	Relationship Energy → Affective music receptivity	0.249	[0.171, 0.331]	Supported
H15	Relationship Energy→ Attentional music receptivity	0.057	[-0.027, 0.142]	Not supported
H16	Affective music receptivity → Performance Self-Efficacy	0.256	[0.156, 0.351]	Supported
H17	Attentional music receptivity → Performance Self-Efficacy	0.130	[0.028, 0.230]	Supported

Specific indirect associations were estimated with 5,000 two-tailed BCa bootstrap samples. Seven of ten intervals excluded zero: reciprocity→affective receptivity→self-efficacy (β = 0.065, 95% BCa CI [0.037, 0.107]); courtesy→affective receptivity→self-efficacy (β = 0.044, [0.022, 0.078]); status→affective receptivity→self-efficacy (β = 0.072, [0.041, 0.113]); energy→affective receptivity→self-efficacy (β = 0.063, [0.037, 0.101]); personality→attentional receptivity→self-efficacy (β = 0.041, [0.008, 0.076]); reciprocity→attentional receptivity→self-efficacy (β = 0.035, [0.008, 0.069]); and status→attentional receptivity→self-efficacy (β = 0.033, [0.008, 0.064]). The other three intervals included zero. These products identify intervening statistical pathways but do not establish temporal mediation. Full results are reported in S3 File.

Predictive relevance was assessed using Q^2^ and PLSpredict. Q^2^ values were 0.225 for attentional music receptivity, 0.276 for affective music receptivity, and 0.310 for performance self-efficacy. For the three attentional indicators, prediction Q^2^ values were 0.354, 0.295, and 0.282; corresponding PLS-based RMSE values (0.512, 0.540, 0.603) were lower than the linear-model benchmarks (0.528, 0.559, 0.624). For the three affective indicators, prediction Q^2^ values were 0.321, 0.274, and 0.233, and PLS RMSE values (0.495, 0.523, 0.601) were lower than the benchmarks (0.513, 0.537, 0.619). For the three self-efficacy indicators, prediction Q^2^ values were 0.276, 0.241, and 0.204, and PLS RMSE values (0.487, 0.544, 0.610) were lower than the benchmarks (0.503, 0.561, 0.637).

The indirect results refine the interpretation of the structural model. The affective route was supported for reciprocity, courtesy, status, and energy, whereas the personality–affective product included zero. The attentional route was supported for personality, reciprocity, and status, whereas the courtesy and energy products included zero. This pattern is consistent with the distinction proposed in the theory section: respectful and energizing interactions correspond more consistently with emotional resonance, while a distinctive persona and recognized relational standing correspond more consistently with focus. Reciprocity was the only relational cue with supported products through both receptivity dimensions.

The effect sizes place these findings in perspective.As shown in Table 4, The direct and indirect coefficients are generally small to moderate, and the model explains 32.7% of attentional receptivity, 38.2% of affective receptivity, and 41.7% of performance self-efficacy. Positive Q^2^ values and favorable PLSpredict comparisons indicate predictive relevance within the observed data. They do not establish transportability to other countries, age groups, or masterclass formats. Replication should evaluate prediction with new samples and record participant role, teacher identity, event format, and prior performance experience.

**Table 4:** Effect sizes (f^2^), explained variance (R^2^), and predictive relevance (Q^2^).

Brand personality	0.126	0.001	0.009		
Courtesy	0.003	0.046	0.066		
Energy	0.004	0.090	0.041		
Reciprocity	0.090	0.089	0.030		
Status	0.086	0.115	0.023		
Affective music receptivity			0.069	0.382	0.276
Attentional music receptivity			0.019	0.327	0.225
Performance Self-Efficacy				0.417	0.310

Note: Predictor-specific f^2^ values are reported in the first three outcome columns; R^2^ and Q^2^ are reported only for the corresponding endogenous constructs.

### 4.3 fsQCA

fsQCA complemented the average net associations from PLS-SEM by examining configurations associated with high performance self-efficacy [23]. Construct means were calibrated using the direct method. The full-membership/crossover/full-nonmembership anchors were: personality 5.000/4.000/2.600; reciprocity 5.000/4.000/2.333; courtesy 5.000/4.000/3.000; status 5.000/4.333/3.000; energy 4.667/3.333/2.000; affective receptivity 5.000/4.000/2.667; attentional receptivity 5.000/4.333/3.000; and performance self-efficacy 5.000/3.667/2.333. The necessity results in Table 5 are interpreted cautiously because several present conditions also had high consistency for low self-efficacy; no single condition is claimed as robustly necessary.

**Table 5:** Analysis of necessary conditions.

Antecedent Condition	High pse		Low pse	
	Consistency	Coverage	Consistency	Coverage
Brand Personality	0.893	0.831	0.944	0.378
~Brand Personality	0.332	0.933	0.578	0.699
Reciprocity	0.913	0.857	0.917	0.37
~Reciprocity	0.329	0.902	0.646	0.761
Courtesy	0.943	0.832	0.958	0.364
~Courtesy	0.279	0.94	0.559	0.807
Status	0.946	0.818	0.945	0.351
~Status	0.25	0.914	0.51	0.802
Energy	0.777	0.912	0.837	0.422
~Energy	0.508	0.879	0.826	0.614
Affective music receptivity	0.93	0.86	0.912	0.362
~Affective music receptivity	0.311	0.891	0.649	0.799
Attentional music receptivity	0.96	0.807	0.973	0.351
~Attentional music receptivity	0.229	0.951	0.467	0.834

The baseline sufficiency analysis used frequency = 1 and raw consistency = 0.80 and yielded seven configurations (Table 6). Because frequency = 1 is permissive for n = 382, robustness analyses used frequency thresholds of 3 and 5, raw consistency thresholds of 0.80 and 0.82, PRI consistency of 0.65, and alternative 90th/50th/10th-percentile calibration anchors. The stricter specifications produced four to five sufficient recipes and lower solution coverage. Full recipes, consistency, coverage, PRI, and calibration results are reported in S3 File; Table 6 presents the baseline values.

**Table 6:** Results of fsQCA.

Brand Personality	•	●	●	●	•		●
Reciprocity		●	●	●		•	●
Courtesy		•	•		•	•	•
Status	•	•		•	•	•	•
Energy	●						●
Affective music receptivity			●	●	●	●	●
Attentional music receptivity	•	•	•	•	•	•	
Raw Coverage	0.714	0.789	0.783	0.789	0.801	0.813	0.451
Unique Coverage	0.007	0.011	0.011	0.007	0.013	0.042	0.002
Consistency	0.948	0.932	0.942	0.938	0.935	0.941	0.967
Solution Coverage	0.899						
Solution Consistency	0.895						

Note. ● indicates core presence; • indicates peripheral presence; blank cells indicate “don’t care” conditions.

Under the baseline solution shown in Table 6, M1 combines the core presence of relationship energy with the peripheral presence of brand personality, relationship status, and attentional music receptivity. Reciprocity, courtesy, and affective music receptivity are “don’t care” conditions in M1. M6 had the largest unique coverage in the baseline solution (0.042), with affective receptivity marked as core and reciprocity, courtesy, status, and attention marked as peripheral. Blank cells indicate conditions that were not required in a recipe, not absent conditions.

The seven baseline configurations should be interpreted as set-theoretic patterns rather than causal effects. Attention, affective receptivity, courtesy, and status recur across several configurations, while personality, reciprocity, and energy are configuration-specific. The available fsQCA outputs did not permit independent reconstruction of every core/peripheral designation because separate parsimonious and intermediate solutions were unavailable. Accordingly, the stricter robustness analyses in S3 File report complete recipes without assigning core/peripheral status where it could not be verified.

Robustness checks reduced the number of identified configurations and lowered solution coverage. The seven baseline recipes should therefore not be treated as the only possible taxonomy of high-self-efficacy masterclass experiences. The more defensible conclusion is that high self-efficacy was associated with multiple multi-condition arrangements in which affective or attentional receptivity frequently participated.

PLS-SEM and fsQCA converge at the level of broad interpretation while differing at the level of individual conditions. PLS-SEM shows which cues have average partial associations after accounting for the other predictors. fsQCA shows that a condition with a weak average association can still be part of a sufficient combination, and that a condition with a positive average association need not be present in every recipe. This complementarity is the principal value of the mixed analytical strategy; it does not license causal claims from cross-sectional data.

## 5 Conclusions, discussion, and implications

Grounded in social cognitive theory, this study examined whether five relational cues in piano masterclasses were associated with performance self-efficacy and with affective and attentional music receptivity. Reciprocity, courtesy, status, and energy were positively associated with self-efficacy; personality was not. Reciprocity, courtesy, status, and energy were associated with affective receptivity, whereas personality, reciprocity, and status were associated with attentional receptivity. Both receptivity dimensions were positively associated with self-efficacy. Seven specific indirect associations had BCa intervals excluding zero. These results identify statistical pathways but do not establish temporal mediation. The baseline fsQCA solution showed equifinality, while stricter specifications yielded fewer configurations and support a cautious emphasis on recurring combinations rather than individual recipes.

The study makes three contributions. First, it clarifies how relationship-interaction-based brand-equity concepts can be adapted as relational learning cues without equating education with consumption. Second, it distinguishes affective and attentional receptivity as related but empirically different correlates of self-efficacy. Third, it combines net-association and configurational perspectives, showing that a nonsignificant average path does not prevent a condition from appearing in some sufficient configurations. The specific indirect estimates provide additional detail on the structural associations, but their cross-sectional nature requires the term “intervening statistical pathway” rather than causal mediation.

Affective and attentional receptivity had different correlates. Courtesy, reciprocity, status, and energy were associated with affective receptivity, whereas personality, reciprocity, and status were associated with attentional receptivity. The fsQCA results add a configurational perspective: no single relational cue was uniformly decisive, and multiple combinations were associated with high self-efficacy. In M1, relationship energy was marked as core, while brand personality, relationship status, and attentional receptivity were marked as peripheral; reciprocity, courtesy, and affective receptivity were “don’t care” conditions. Because the seven baseline configurations were not recovered under all stricter specifications, interpretation focuses on recurring combinations rather than treating any one recipe as definitive.

The distinction between affective and attentional receptivity also advances interpretation of the masterclass as a public learning setting. [8–10] shows that performers and observers encounter related but different learning opportunities: performers receive public coaching and concert-like practice, whereas observers must select, compare, and transfer what they see and hear. The present results add a perceptual pathway to that account. Emotional resonance was associated particularly with encouragement, respect, reciprocity, and relational vitality, while sustained attention was associated particularly with a salient teacher, worthwhile exchange, and perceived recognition. These associations suggest why merely being present in the audience may be insufficient; the relational conditions of the episode may shape whether observation becomes emotionally or cognitively receptive. Because the survey did not record whether respondents were the performer or an observer, these role-specific interpretations remain theoretical and should be tested directly.

The nonsignificant direct association between personality and self-efficacy is theoretically informative [8,10] notes that a renowned master can embody professional authority and confer symbolic status, yet reputation alone does not ensure that useful learning occurs. The present pattern is consistent with that distinction: prestige, distinctiveness, and artistic persona were associated with attention but not directly with feeling more capable. The supported personality–attention–self-efficacy product is compatible with the possibility that persona first makes the demonstration salient, although the cross-sectional data cannot establish that temporal sequence. Masterclass organizers should therefore distinguish visibility from pedagogy and support renowned teachers with interaction structures that make their reasoning, modelling, and feedback accessible.

Reciprocity occupies a different position because it was associated with both forms of receptivity and with self-efficacy, and both specific indirect products excluded zero. In this context, reciprocity means perceived educational return: participants believe that the attention, guidance, and experience received are worthwhile relative to their investment. The result is compatible with a learning environment in which participants attend because the exchange is valuable and respond emotionally because they feel recognized. Longitudinal research is needed to establish whether changes in perceived reciprocity precede changes in receptivity and confidence.

The findings suggest cautious implications for masterclass design. Teachers and organizers can foster respectful communication, useful reciprocal feedback, clear recognition of participants, and emotionally engaging demonstrations. Listening prompts may help observers turn a public masterclass into deliberate learning, while staged performance tasks may provide performers with attainable mastery experiences. These recommendations are consistent with the observed associations but should not be read as proven intervention effects. Future longitudinal or experimental studies should test whether changes in feedback, observation guidance, or participation structure precede changes in receptivity and self-efficacy.

For performers, respectful diagnostic feedback can be organized around observable musical decisions rather than global judgments of talent. Teachers can identify a technical or interpretive problem, model an alternative, invite another attempt, and explicitly mark what improved. For observers, brief listening prompts can direct attention to phrasing, articulation, coordination, tone, or practice strategy before and after the public coaching episode. This recommendation follows Haddon’s [24] analysis of observational learning and [9,10] argument that audience learning becomes stronger when students actively observe, compare, assess, reflect, and connect the episode with their own practice.

Organizers can also clarify the relational referent of the event. Publicity may emphasize the teacher’s artistic identity, but the learning design should provide reciprocal value through accessible explanations, respectful interaction, and opportunities for participants to connect demonstrations with their own practice. Recognition can involve addressing participant questions seriously and acknowledging different experience levels. These practices are plausible applications of the findings, not tested interventions.

This study has several limitations. First, the cross-sectional, single-wave design does not establish temporal order, causality, or mediation; reverse and reciprocal associations remain possible. Second, retrospective reports about one masterclass may be affected by recall and common-method variance, although full-collinearity VIFs were below 3.3. Third, convenience recruitment produced a predominantly adult, highly educated, music-trained sample with a near-even gender distribution, limiting generalization to children, routine conservatoire cohorts, and the wider population of Chinese piano learners. Fourth, the questionnaire did not distinguish whether respondents participated primarily as performers or observers. Fifth, respondents were instructed to evaluate the pianist/master teacher, but some may still have incorporated the series or host institution. Sixth, the baseline fsQCA solution was sensitive to stricter frequency and calibration specifications, and complete parsimonious and intermediate solution outputs were unavailable for independently verifying every core/peripheral designation. Future research should distinguish participant roles, preregister the focal referent and adaptation process, and use longitudinal, experimental, or repeated-measures designs.

Future longitudinal work could separate the sequence implied by the present model. Relational cues could be measured before a masterclass, receptivity during or immediately after the event, and self-efficacy before and after a subsequent performance task. Repeated measurements would allow researchers to examine within-person change and reciprocal relationships rather than relying only on between-person covariance. An experimental extension could vary feedback, observation guidance, or opportunities for interaction while holding repertoire and teacher constant.

Measurement development is another priority. Although the present study used contextual adaptation, forward and back translation for English-language items, expert review, and pilot testing, those procedures do not by themselves establish measurement invariance across participant groups. Future studies should conduct cognitive interviews with performers and observers to examine how they understand “this pianist/master teacher,” reciprocity, status, and energy, and should test invariance across age, role, and professional background. The three self-efficacy items should also be compared with longer music-performance measures and behavioral indicators such as voluntary performance, persistence after feedback, or judged performance quality.

Configurational replication should distinguish exploratory from confirmatory use of fsQCA. Calibration anchors and truth-table thresholds can be prespecified, and robustness can be evaluated across reasonable alternatives before naming configurations. New samples could test whether the recurring combinations observed here show comparable consistency and coverage. Reporting the truth table, contradictory rows, PRI, intermediate solution, parsimonious solution, and directional expectations would permit independent reconstruction of core and peripheral conditions.

The present data nevertheless provide a useful baseline for that program of research. The sample includes variation in age, educational attainment, and recalled masterclass experience, and the anonymized item-level file permits independent checking of the supplementary diagnostics. The interpretation distinguishes the empirical findings (reliable measures, differentiated associations, indirect products, predictive relevance, and sensitivity to configuration choices) from questions the design cannot resolve (temporal ordering, intervention effectiveness, or universal recipes).

## Supporting information

S1 Data Anonymized survey dataset. (XLSX)

S2 File Complete bilingual questionnaire. (DOCX)

S3 File Statistical supplement. (DOCX)
