# Supplementary material for: Relational learning cues, music receptivity, and performance self-efficacy among piano masterclass participants: A PLS-SEM and fsQCA study
Source: PLoS One. 2026 Nov 3;21(11):e0361767. doi: 10.1371/journal.pone.0361767 (PMC13730353; doi:10.1371/journal.pone.0361767)
Supplement: Supplementary file 1 [file pone.0361767.s002.docx]

S2 File. Complete Bilingual Questionnaire

Instruction used for analysis: Please recall one specific piano masterclass and answer with reference to the pianist/master teacher in that experience. Response scale for all items: 1 = strongly disagree; 2 = disagree; 3 = neither agree nor disagree; 4 = agree; 5 = strongly agree.

Note. The Chinese wording reproduces the administered items. The English column provides a faithful translation for reporting and was not separately administered.

| **Code** | **Construct** | **Administered Chinese item** | **Faithful English translation** | **Source/adaptation** |
| --- | --- | --- | --- | --- |
| BP1 | Brand personality | 这位钢琴大师的艺术风格是独特的。 | This pianist/master teacher has a distinctive artistic style. | Zhong & Zhang (2015); contextual adaptation |
| BP2 | Brand personality | 我认为这位钢琴大师在音乐界享有较高的地位。 | I believe this pianist/master teacher has high professional standing in the music field. | Zhong & Zhang (2015); contextual adaptation |
| BP3 | Brand personality | 我认为这位钢琴大师知名度较高。 | I believe this pianist/master teacher is widely recognized. | Zhong & Zhang (2015); contextual adaptation |
| BP4 | Brand personality | 我认为这位钢琴大师有较好的声誉。 | I believe this pianist/master teacher has a good reputation. | Zhong & Zhang (2015); contextual adaptation |
| BP5 | Brand personality | 这位钢琴大师的个人风格让我印象深刻。 | This pianist/master teacher’s personal style leaves a strong impression on me. | Zhong & Zhang (2015); contextual adaptation |
| REC1 | Reciprocity | 我觉得这位钢琴大师带给我的收获很大。 | I gained substantial benefits from this pianist/master teacher. | Zhong & Zhang (2015); contextual adaptation |
| REC2 | Reciprocity | 相对于其他老师相比，这位老师能给我带来较多的收获。 | Compared with other teachers, this pianist/master teacher brings me greater benefits. | Zhong & Zhang (2015); contextual adaptation |
| REC3 | Reciprocity | 这位大师带给我的收获值得我的付出。 | The benefits I gain from this pianist/master teacher are worth my effort and investment. | Zhong & Zhang (2015); contextual adaptation |
| COU1 | Courtesy | 这位钢琴大师对待学员非常友好。 | This pianist/master teacher is very friendly toward participants. | Zhong & Zhang (2015); contextual adaptation |
| COU2 | Courtesy | 这位钢琴大师比较关心学员。 | This pianist/master teacher cares about participants. | Zhong & Zhang (2015); contextual adaptation |
| COU3 | Courtesy | 这位钢琴大师比较尊重学员。 | This pianist/master teacher treats participants with respect. | Zhong & Zhang (2015); contextual adaptation |
| STA1 | Relationship status | 我觉得这位钢琴大师的形象是可靠的。 | I perceive this pianist/master teacher as reliable. | Zhong & Zhang (2015); contextual adaptation |
| STA2 | Relationship status | 我认为这位钢琴大师是值得信赖的。 | I believe this pianist/master teacher is trustworthy. | Zhong & Zhang (2015); contextual adaptation |
| STA3 | Relationship status | 我觉得这位大师的专业能力可以给学生带来安全感 | This pianist/master teacher’s professional ability gives participants a sense of security and dependability. | Zhong & Zhang (2015); contextual adaptation |
| ENE1 | Relationship energy | 我经常关注这位钢琴大师。 | I often follow this pianist/master teacher’s activities. | Zhong & Zhang (2015); contextual adaptation |
| ENE2 | Relationship energy | 我愿意付出时间和金钱来购买这位钢琴大师的课程。 | I am willing to invest time and money in this pianist/master teacher’s courses. | Zhong & Zhang (2015); contextual adaptation |
| ENE3 | Relationship energy | 我愿意在未来继续支持和关注这位钢琴大师。 | I intend to continue supporting and following this pianist/master teacher. | Zhong & Zhang (2015); contextual adaptation |
| AFF1 | Affective receptivity | 在上这位钢琴家举办的课堂过程中，他教授的曲子能让我产生情感共鸣。 | During this pianist/master teacher’s masterclass, the music being taught evokes emotional resonance in me. | George & Ilavarasu (2021); contextual adaptation |
| AFF2 | Affective receptivity | 在上这位钢琴家举办的课堂过程中，我会受到音乐的情感触动。 | During this pianist/master teacher’s masterclass, I am emotionally moved by the music. | George & Ilavarasu (2021); contextual adaptation |
| AFF3 | Affective receptivity | 在上这位钢琴家举办的课堂过程中，我会产生感动、激动、愉悦等情感体验。 | During this pianist/master teacher’s masterclass, I experience feelings such as being moved, excited, or pleased. | George & Ilavarasu (2021); contextual adaptation |
| ATT1 | Attentional receptivity | 他教授曲子的时候，我的注意力比较集中。 | When this pianist/master teacher teaches a piece, I can concentrate well. | George & Ilavarasu (2021); contextual adaptation |
| ATT2 | Attentional receptivity | 大师示范乐曲时，我的认知专注度维持在较高水平。 | When the master demonstrates a piece, I maintain a high level of cognitive focus. | George & Ilavarasu (2021); contextual adaptation |
| ATT3 | Attentional receptivity | 他教授曲子的时候，可以持续地吸引着我的关注。 | When this pianist/master teacher teaches a piece, my attention remains engaged. | George & Ilavarasu (2021); contextual adaptation |
| PSE1 | Performance self-efficacy | 在参加这个大师的课程中，我相信自己能够演奏好。 | In this masterclass, I am confident that I can perform well. | Romppel et al. (2013); contextual adaptation |
| PSE2 | Performance self-efficacy | 我对自己演奏好大师教授的曲目充满信心。 | I am confident that I can perform the repertoire taught by the master well. | Romppel et al. (2013); contextual adaptation |
| PSE3 | Performance self-efficacy | 即使作品难度较高，我也相信自己能克服困难。 | Even when a piece is difficult, I believe I can overcome the challenges. | Romppel et al. (2013); contextual adaptation |
