# Supplementary material for: Relational learning cues, music receptivity, and performance self-efficacy among piano masterclass participants: A PLS-SEM and fsQCA study
Source: PLoS One. 2026 Nov 3;21(11):e0361767. doi: 10.1371/journal.pone.0361767 (PMC13730353; doi:10.1371/journal.pone.0361767)
Supplement: Supplementary file 2 [file pone.0361767.s003.docx]

S3 Statistical Supplement

This supplement reports additional diagnostics and robustness analyses calculated from the anonymized item-level dataset. These calculations supplement the retained PLS-SEM and fsQCA results and are reported to make the revision independently auditable.

**Table 1. HTMT discriminant-validity ratios**

|  | **ATT** | **BP** | **COU** | **AFF** | **ENE** | **PSE** | **REC** | **STA** |
| --- | --- | --- | --- | --- | --- | --- | --- | --- |
| ATT |  |  |  |  |  |  |  |  |
| BP | 0.481 |  |  |  |  |  |  |  |
| COU | 0.150 | 0.261 |  |  |  |  |  |  |
| AFF | 0.297 | 0.251 | 0.367 |  |  |  |  |  |
| ENE | 0.285 | 0.242 | 0.129 | 0.476 |  |  |  |  |
| PSE | 0.403 | 0.182 | 0.426 | 0.621 | 0.444 |  |  |  |
| REC | 0.472 | 0.240 | 0.273 | 0.504 | 0.290 | 0.490 |  |  |
| STA | 0.444 | 0.185 | 0.169 | 0.502 | 0.261 | 0.442 | 0.260 |  |

Note. All HTMT ratios are below 0.90.

**Table 2. Full-collinearity VIFs**

| **Construct** | **VIF** |
| --- | --- |
| Brand personality | 1.274 |
| Reciprocity | 1.422 |
| Courtesy | 1.222 |
| Relationship status | 1.366 |
| Relationship energy | 1.281 |
| Affective music receptivity | 1.734 |
| Attentional music receptivity | 1.523 |
| Performance self-efficacy | 1.712 |

Note. All values are below the 3.3 diagnostic threshold. SRMR = 0.047.

**Table 3. Bootstrapped specific indirect associations**

| **Specific indirect path** | **β** | **95% BCa CI** | **CI excludes zero** |
| --- | --- | --- | --- |
| BP → AFF → PSE | .007 | [−.017, .031] | No |
| REC → AFF → PSE | .065 | [.036, .106] | Yes |
| COU → AFF → PSE | .043 | [.022, .076] | Yes |
| STA → AFF → PSE | .071 | [.041, .113] | Yes |
| ENE → AFF → PSE | .063 | [.036, .101] | Yes |
| BP → ATT → PSE | .040 | [.008, .076] | Yes |
| REC → ATT → PSE | .035 | [.008, .070] | Yes |
| COU → ATT → PSE | −.006 | [−.025, .004] | No |
| STA → ATT → PSE | .033 | [.008, .065] | Yes |
| ENE → ATT → PSE | .007 | [−.002, .025] | No |

Note. The estimates use standardized unit-weighted construct scores, 5,000 bootstrap resamples, two-tailed 95% bias-corrected and accelerated confidence intervals, jackknife-derived acceleration, and fixed seed 20260824. They are supplementary cross-sectional indirect associations, not recovered SmartPLS specific-indirect-effects output and not evidence of temporal mediation.

**Table 4. Empirical fsQCA calibration anchors**

| **Set** | **Full membership (95th)** | **Crossover (50th)** | **Full non-membership (5th)** |
| --- | --- | --- | --- |
| BP | 5.000 | 4.000 | 2.600 |
| REC | 5.000 | 4.000 | 2.333 |
| COU | 5.000 | 4.000 | 3.000 |
| STA | 5.000 | 4.333 | 3.000 |
| ENE | 4.667 | 3.333 | 2.000 |
| AFF | 5.000 | 4.000 | 2.667 |
| ATT | 5.000 | 4.333 | 3.000 |
| PSE | 5.000 | 3.667 | 2.333 |

**Table 5. fsQCA robustness checks**

| **Specification** | **Thresholds** | **Configurations** | **Solution consistency** | **Solution coverage** | **Interpretation** |
| --- | --- | --- | --- | --- | --- |
| Baseline reported in main manuscript | Frequency 1; consistency .80 | 7 | .895 | .899 | Original Table 6 retained unchanged |
| Higher empirical frequency | Frequency 3; consistency .80; PRI .65 | 5 | .927 | .535 | Fewer, more conservative configurations |
| Stricter frequency | Frequency 5; consistency .80; PRI .65 | 4 | .938 | .460 | Fewer, more conservative configurations retained |
| Higher consistency | Frequency 3; consistency .82; PRI .65 | 5 | .927 | .535 | Same as frequency-3 solution |
| Alternative calibration | 90th/50th/10th percentiles; frequency 3 | 5 | .928 | .440 | Relational cues and affective receptivity remained prominent |

The stricter specifications do not reproduce all seven baseline configurations. Accordingly, the main-text discussion treats individual configurations cautiously and emphasizes only recurring multi-condition patterns.
