# Supplementary material for: Relational learning cues, music receptivity, and performance self-efficacy among piano masterclass participants: A PLS-SEM and fsQCA study
Source: PLoS One. 2026 Nov 3;21(11):e0361767. doi: 10.1371/journal.pone.0361767 (PMC13730353; doi:10.1371/journal.pone.0361767)
Supplement: Supplementary file 3 [file pone.0361767.s004.docx]

**Response to the Academic Editor and Reviewers**

**Manuscript ID: PONE-D-26-28600**

Dear Academic Editor and Reviewers,

Thank you for the careful and constructive review of our manuscript. We revised the paper and its supplementary files point by point. Our responses below explain what was changed and where the revised materials provide the requested evidence.

## 1. Editorial requirements: title page, ethics, consent, and data availability

**Response:** A complete title page is included. Methods now identifies the Ethics Review Committee of Qilu Normal University, approval date January 23, 2025, and Ethical Approval No. xsllsc2025-028. Adults clicked “Agree” to provide electronic written consent; a parent or legal guardian used the same recorded procedure for participants younger than 18. S1 Data, S2 File, and S3 Statistical Supplement are identified in the Data Availability Statement.

## 2. Reviewer 1: clarify the focal “brand”

**Response:** The focal referent is now explicitly defined as the pianist/master teacher in one specific recalled masterclass. The manuscript also acknowledges that some respondents may have incorporated the series or host institution.

## 3. Reviewer 1: citation audit

**Response:** Clearly mismatched or decorative citations were removed or replaced. The five-dimensional relationship-interaction scale is attributed to Zhong and Zhang (2015), and unsupported citations at H1, H2, and H6 were removed. The reference list itself was otherwise preserved and supplemented with directly relevant sources.

## 4. Reviewer 1: add masterclass scholarship

**Response:** The Introduction now discusses public masterclasses, observational learning, and the differing learning positions of performers and observers, citing Haddon (2014) and Hanken (2015).

## 5. Reviewer 1: performer versus observer

**Response:** Because the questionnaire did not record role, the manuscript explicitly identifies this as a limitation and does not retrospectively classify participants.

## 6. Reviewer 1: sample framing and income

Response: We removed the adult-only label from the title because the sample included both adults and adolescents. The abstract now describes 382 adult and adolescent participants, and the Methods reports the complete age, education, gender, and music-background distributions. We also added a limitation concerning the predominantly adult, highly educated, music-trained sample. References to income were removed because income variables are not present in the retained analytic dataset.

## 7. Reviewer 1 and Reviewer 2: measurement and translation

**Response:** Methods now specifies the five Likert response points, construct-level sources, contextual adaptation, and the questionnaire-development procedure. For the English-language receptivity and self-efficacy items, a bilingual researcher produced the Chinese translation, a second bilingual researcher independently back-translated it, and discrepancies were resolved against the source wording. Reviewers familiar with music education and questionnaire design assessed semantic equivalence, clarity, and contextual relevance. The questionnaire was then pilot-tested with people who had piano-masterclass experience; ambiguous wording was refined, and pilot responses were excluded from the analytic sample. S2 File provides all 26 administered Chinese items, faithful English translations, construct sources, and adaptation notes.

## 8. Reviewer 1: common-method bias

**Response:** S3 Table 2 reports full-collinearity VIFs for all eight constructs (1.222–1.735), all below 3.3.

## 9. Reviewer 1: PLS-SEM reporting completeness

**Response:** Methods now reports 5,000 bootstrap resamples, two-tailed tests, 95% BCa intervals, HTMT, Fornell–Larcker, SRMR = 0.047, and inverse-square-root sample-size reasoning. Table 3 retains every original number; its caption and note now distinguish predictor-specific f² from endogenous-construct R² and Q². Complete additional diagnostics are in S3.

## 10. Reviewer 1 and Reviewer 2: specific indirect effects

Response: We estimated all ten specific indirect associations with 5,000 two-tailed BCa bootstrap samples; seven confidence intervals excluded zero. S3 Table 3 reports every estimate and interval. Throughout the abstract, hypotheses, results, and discussion, we use “specific indirect association” or “intervening statistical pathway.” We no longer describe music receptivity as a demonstrated mediator, because the cross-sectional design cannot establish temporal mediation.

## 11. Reviewer 1: fsQCA threshold, anchors, PRI, core/peripheral logic, and robustness

Response: The main manuscript reports all empirical calibration anchors, the PRI criterion, and the parsimonious/intermediate nesting rule. To preserve the validated original result tables, we retained Tables 5–6 in their original form. S3 Table 5 transparently compares that baseline with frequency thresholds of 3 and 5, consistency .82, PRI .65, and alternative calibration. The stricter solutions do not reproduce all seven baseline configurations; we now discuss this sensitivity explicitly.

## 12. Reviewer 1: configuration-description mismatch

Response: We corrected the Results and Discussion so that M1 is described as the complete configuration displayed in Table 6, explicitly acknowledging courtesy and affective receptivity. We removed the earlier selective description that reduced M1 to relationship energy, brand personality, relationship status, and attentional receptivity.

## 13. Reviewer 1 and Reviewer 2: causal language

Response: We searched the full manuscript for “influence,” “affect,” “effect,” “lead to,” “enhance,” “cause,” and “causal.” Statements about our findings in the hypotheses, Methods, Results, fsQCA section, and Discussion now use noncausal language such as “associated with,” “positively related to,” “specific indirect association,” and “configuration associated with.” The limitations explicitly address the cross-sectional design, retrospective recall, convenience sampling, and possible reverse or reciprocal relationships.

## 14. Reviewer 1: PLOS-specific issues

**Response:** The ethics relationship is explained: Qilu Normal University provided local oversight because recruitment and data collection occurred in China, whereas the Moscow affiliations are academic appointments. Corresponding-author location must be set manually in Editorial Manager to Saint Petersburg, Russia.

## 15. Reviewer 1: formatting

Response: We corrected the duplicate table numbering. The tables are now numbered consecutively from Table 1 through Table 6, and the reference-list heading is now “References.” We also standardized spacing, Q² notation, caption wording, et al. usage, and American English. Tables remain in a plain black-and-white format.

# Files included with the revision

Clean revised manuscript

Highlighted revised manuscript

S1 Data: anonymized analytic dataset

S2 File: complete bilingual questionnaire

S3 Statistical Supplement: HTMT, VIF, SRMR, specific indirect associations, calibration anchors, and fsQCA robustness checks

Ethics approval certification

We appreciate the reviewers’ guidance. The revision has helped us clarify the study’s scope, report the supplementary analyses more transparently, and align the interpretation with what the cross-sectional data can support.

Sincerely,
Zhao Yixuan, Yan Su, and Zhu Xiaohui
