# Supplementary material for: Relational learning cues, music receptivity, and performance self-efficacy among piano masterclass participants: A PLS-SEM and fsQCA study
Source: PLoS One. 2026 Nov 3;21(11):e0361767. doi: 10.1371/journal.pone.0361767 (PMC13730353; doi:10.1371/journal.pone.0361767)
Supplement: Peer review history

# Peer review: Decision Letter 0

**DOI:** 10.1371/journal.pone.0361767.r001

**Authors:** Chao Cheng

19 Aug 2026

Relational Learning Cues, Music Receptivity, and Performance Self-Efficacy Among Piano Masterclass Students: A PLS-SEM and fsQCA Study

PLOS One

Dear Dr.  Zhu,

Thank you for submitting your manuscript to PLOS ONE. After careful consideration, we feel that it has merit but does not fully meet PLOS ONE’s publication criteria as it currently stands. Therefore, we invite you to submit a revised version of the manuscript that addresses the points raised during the review process.

Please submit your revised manuscript by Oct 18 2026 11:59PM. If you will need more time than this to complete your revisions, please reply to this message or contact the journal office at plosone@plos.org. When you're ready to submit your revision, log on to https://www.editorialmanager.com/pone/ and select the 'Submissions Needing Revision' folder to locate your manuscript file.

Please include the following items when submitting your revised manuscript:

- A letter that responds to each point raised by the academic editor and reviewer(s). You should upload this letter as a separate file labeled 'Response to Reviewers'.
- A marked-up copy of your manuscript that highlights changes made to the original version. You should upload this as a separate file labeled 'Revised Manuscript with Track Changes'.
- An unmarked version of your revised paper without tracked changes. You should upload this as a separate file labeled 'Manuscript'.

If you would like to make changes to your financial disclosure, please include your updated statement in your cover letter. Guidelines for resubmitting your figure files are available below the reviewer comments at the end of this letter.

If applicable, we recommend that you deposit your laboratory protocols in protocols.io to enhance the reproducibility of your results. Protocols.io assigns your protocol its own identifier (DOI) so that it can be cited independently in the future. For instructions see: https://journals.plos.org/plosone/s/submission-guidelines#loc-laboratory-protocols. Additionally, PLOS ONE offers an option for publishing peer-reviewed Lab Protocol articles, which describe protocols hosted on protocols.io. Read more information on sharing protocols at https://plos.org/protocols?utm_medium=editorial-email&utm_source=authorletters&utm_campaign=protocols.

As the corresponding author, your ORCID iD is verified in the submission system and will appear in the published article. PLOS supports the use of ORCID, and we encourage all coauthors to register for an ORCID iD and use it as well. Please encourage your coauthors to verify their ORCID iD within the submission system before final acceptance, as unverified ORCID iDs will not appear in the published article. *Only*  the individual author can complete the verification step; PLOS staff *cannot*  verify ORCID iDs on behalf of authors.

We look forward to receiving your revised manuscript.

Kind regards,

Chao Cheng

Guest Editor

PLOS One

Journal Requirements:

When submitting your revision, we need you to address these additional requirements.

1.Please ensure that your manuscript meets PLOS ONE's style requirements, including those for file naming. The PLOS ONE style templates can be found at

https://journals.plos.org/plosone/s/file?id=wjVg/PLOSOne_formatting_sample_main_body.pdf and

https://journals.plos.org/plosone/s/file?id=ba62/PLOSOne_formatting_sample_title_authors_affiliations.pdf

2. Please provide additional details regarding participant consent. In the ethics statement in the Methods and online submission information, please ensure that you have specified (1) whether consent was informed and (2) what type you obtained (for instance, written or verbal, and if verbal, how it was documented and witnessed). If your study included minors, state whether you obtained consent from parents or guardians. If the need for consent was waived by the ethics committee, please include this information.

If you are reporting a retrospective study of medical records or archived samples, please ensure that you have discussed whether all data were fully anonymized before you accessed them and/or whether the IRB or ethics committee waived the requirement for informed consent. If patients provided informed written consent to have data from their medical records used in research, please include this information.

3. In the online submission form you indicate that your data is not available for proprietary reasons and have provided a contact point for accessing this data. Please note that your current contact point is a co-author on this manuscript. According to our Data Policy, the contact point must not be an author on the manuscript and must be an institutional contact, ideally not an individual. Please revise your data statement to a non-author institutional point of contact, such as a data access or ethics committee, and send this to us via return email. Please also include contact information for the third party organization, and please include the full citation of where the data can be found.

4. Please ensure that you include a title page within your main document. You should list all authors and all affiliations as per our author instructions and clearly indicate the corresponding author.

5. Please include your full ethics statement in the ‘Methods’ section of your manuscript file. In your statement, please include the full name of the IRB or ethics committee who approved or waived your study, as well as whether or not you obtained informed written or verbal consent. If consent was waived for your study, please include this information in your statement as well.

6. We note you have included a table to which you do not refer in the text of your manuscript. Please ensure that you refer to Table 1-3 in your text; if accepted, production will need this reference to link the reader to the Table.

7. If the reviewer comments include a recommendation to cite specific previously published works, please review and evaluate these publications to determine whether they are relevant and should be cited. There is no requirement to cite these works unless the editor has indicated otherwise.

[Note: HTML markup is below. Please do not edit.]

Reviewers' comments:

Reviewer's Responses to Questions

**Comments to the Author**

1. Is the manuscript technically sound, and do the data support the conclusions?

Reviewer #1: Yes

Reviewer #2: Yes

**********

2. Has the statistical analysis been performed appropriately and rigorously?

Reviewer #1: Yes

Reviewer #2: Yes

**********

3. Have the authors made all data underlying the findings in their manuscript fully available?

The PLOS Data policy

Reviewer #1: Yes

Reviewer #2: Yes

**********

4. Is the manuscript presented in an intelligible fashion and written in standard English?

Reviewer #1: Yes

Reviewer #2: Yes

**********

Reviewer #1: Reviewers’ Comments to the Authors

Abstract. This paper reports on a cross-sectional survey of 382 respondents in mainland China who had participated in piano masterclasses. The study aimed to examine whether the five dimensions of “interactive brand equity” are associated with performance self-efficacy—both directly and indirectly through emotional and attentional musical receptivity. The study employed the PLS-SEM method, supplemented by fsQCA analysis. The concept of viewing master classes as a relational, reputation-oriented learning environment is indeed quite novel, and the authors demonstrate great candor in addressing issues that many tend to avoid—such as a cautious interpretation of the necessity analysis and the explicit acknowledgment that certain indirect effects were not tested using the self-help method. The measurement model is appropriate, and the overall analytical strategy is sound. I recommend B minor revisionsB ; most of the points discussed below pertain to precision of wording, completeness of reporting, and adherence to academic conventions, rather than the substance of the research.

Please clarify what is meant by “brand.” Throughout the paper, it is never explicitly stated whether participants were evaluating the lead instructor, the masterclass series/program, or the organizing institution. These are distinct entities, and the interpretation of each brand equity dimension depends on the specific entity the respondents had in mind. Please clearly specify the exact instructions or definitions presented to participants, add them to the “Methods” section, and note in the “Limitations” section that the referent may vary among respondents.

Reference Review (Most Important Revision). Some in-text citations fail to support the arguments to which they are attached, and several citations at the end of hypotheses appear to serve only a decorative purpose. Examples: The definition of “brand reciprocity” cites the study by Affuso et al. (2023), which examines teacher support and parental supervision; the definition of “brand politeness” cites the study by Luo et al. (2023), which examines learning engagement and academic achievement; “Heuristic teaching” is attributed to the study by Locke and Latham (2002), whereas that study actually pertains to goal-setting theory; the argument that “prestigious master classes operate through reputation and relational continuity” cites Bandura (1997); Keller (1993) is cited as “interactive brand equity,” although this construct, in this form, belongs to Zhang et al. (2020) ; Furthermore, H1, H2, and H6 are each cited with McPherson & McCormick (2006), Høffding et al. (2024), and McCormick & McPherson (2003) as references, respectively, but these sources are unrelated to the stated hypotheses. Please verify each in-text citation and its source, and replace or delete them as appropriate.

Please refer to the relevant literature on master classes. For a paper premised on the idea that “a master class is a unique relational field,” the music education literature on master classes—such as teacher-student interactions and learning through observation of public performances (e.g., Hanken’s study on student learning in master classes)—is almost entirely absent; the research framework relies almost entirely on consumer research. Adding one or two paragraphs to situate this study within the relevant literature would help strengthen the introduction and is also crucial for interpreting the arguments I will present below.

Performer or Observer? The “master class experience” can refer either to performing under guidance or to sitting in the audience, and the sources of self-efficacy involved—experiences of mastery versus indirect experiences—differ fundamentally. If the questionnaire distinguished between these roles, please report their distribution and conduct comparisons or controls; if not, this must be explicitly acknowledged as a limitation of the study, and related arguments should be qualified accordingly.

Please align the sample with the “student” framework. Nearly half of the respondents were aged 26–30, another 42% were aged 31–40, and 44.5% held a master’s degree—these are primarily adult learners and professionals, rather than “students” in the traditional conservatory sense. Please describe the study population more accurately (if necessary, in the title or abstract) and discuss how this composition affects the generalizability of the findings. The slightly higher proportion of men (51.8%) is also atypical among piano learners in China; a specific note regarding its representativeness should be included. Finally, the text mentions that data on income sources and levels were collected but are not presented in the main text; please either report this information or remove the reference.

Measurement Report. The scale points for the scales have never been explicitly stated. Please report them and provide the complete item wording (in both Chinese and English) as supplementary material, while also describing the translation/adaptation procedure and clearly indicating which source scale each construct item was adapted from—the current placement of citations to Börekci et al. and Ou & Qin makes this unclear.

Common methodological biases. Although the procedural remedies are appropriate, statistical tests should be conducted for a single-source, single-wave survey—complete VIF values for multicollinearity would suffice; please report these.

PLS-SEM reporting completeness. Please supplement the guidance on the settings (number of resamples, one-tailed vs. two-tailed tests, BCa), the HTMT ratio, and include the Fornell–Larcker index, the SRMR index, and a brief sample size justification (e.g., the root-mean-square method). Table 3 also needs to be restructured: currently, f², R², and Q² are crammed into the same table and lack clear headings.

Testing indirect effects. The authors honestly note that they did not conduct Bootstrap tests for specific indirect effects, but the term “passed” is repeatedly used in the abstract and discussion—a term specific to mediation analysis. Given that no new data is required for this—SmartPLS reports specific indirect effects with bias-corrected confidence intervals—please supplement the table and provide an interpretation; if this is not possible, please remove all references to mediation analysis from the entire manuscript (including the abstract).

fsQCA details. With a sample size of n = 382, setting the frequency threshold to 1 is too low by current standards; please explain your rationale or raise this threshold, and report whether the results change. Please also report the actual calibration anchor values for each variable, PRI consistency, the derivation methods for core and peripheral states (simplified method/intermediate nesting method), and at least one robustness test (alternative anchor values or consistency thresholds). Furthermore, the textual description of the M1 configuration in the “Discussion” section clearly contradicts Table 6—the table includes “politeness” and “emotional acceptability,” but these terms are not mentioned in the main text; please verify the description of each configuration against the table.

Causal language. This study employs a cross-sectional and retrospective design, yet the Hypotheses and “Results” sections use terms such as “affect” and “lead to.” Please consistently use correlational language (as is largely done in the abstract) and include “cross-sectional design,” “retrospective recall of ‘a specific masterclass,’” and “convenience sampling” in the discussion of study limitations.

PLOS-specific requirements. (a) “Available upon reasonable request” generally does not comply with the PLOS ONE data policy; please deposit anonymized data and survey materials in a data repository, or specify any ethical/legal restrictions. (b) The ethics review board is Qilu Normal University, yet the listed affiliated institution is Moscow State Pedagogical University; adding a sentence explaining the relationship between the two would prevent future questions. (c) The submission metadata lists the corresponding author’s location as “St. Petersburg, Saint-Pierre and Miquelon”—please correct this.

Formatting and Typos (non-exhaustive list). Two tables are both numbered “Table 1”; the heading “Reference” should be changed to “References”; missing spaces (e.g., “(Bandura, 1997).In…”); Panadero (2023), Ahmetović (2020), and Gómez (2016) should be changed to “et al.”; Inconsistent use of “Q2” and “Q²”; brand personality is defined twice, in the “Introduction” and in Section 2.2; British and American spellings are mixed; furthermore, several statements in the “Introduction” regarding previous research findings (e.g., “one-on-one coaching can boost self-confidence”) are not cited.

Reviewer #2: The manuscript addresses an interesting topic and provides a useful interdisciplinary perspective on performance self-efficacy in piano masterclasses. The combination of PLS-SEM and fsQCA is generally appropriate, and the findings are potentially valuable. However, several issues concerning conceptual clarity, mediation interpretation, causal language, and scale adaptation should be clarified before publication. I therefore recommend minor revision.

1.The conceptualization of interactive brand equity in the piano-masterclass context should be clarified. In particular, the manuscript should clearly specify whether the “masterclass brand” refers to the individual instructor, the organizing institution, or the masterclass program. In addition, the definitions of constructs such as relationship status and relationship energy should remain consistent throughout the manuscript.

2.The manuscript frequently describes music receptivity as a mediator, although specific indirect effects were not formally tested. The authors are encouraged either to report bootstrapped indirect effects or to revise the terminology and describe music receptivity more cautiously as an intervening or associated psychological pathway.

3.Given the cross-sectional survey design, causal expressions such as “influences,” “enhances,” and “leads to” should be used more cautiously. The authors should preferably use terms such as “is associated with” or “is positively related to” and briefly acknowledge the possibility of reverse or reciprocal relationships.

4.More information should be provided regarding the adaptation and translation of the measurement scales. The authors should briefly explain how the original items were adapted to the piano-masterclass context and whether procedures such as translation/back-translation, expert review, or pilot testing were conducted. Providing the complete measurement items in the supplementary material would also improve transparency.

**********

what does this mean?). If published, this will include your full peer review and any attached files.

If you choose “no”, your identity will remain anonymous but your review may still be made public.

**Do you want your identity to be public for this peer review?**  For information about this choice, including consent withdrawal, please see our Privacy Policy

Reviewer #1: No

Reviewer #2: No

**********

[NOTE: If reviewer comments were submitted as an attachment file, they will be attached to this email and accessible via the submission site. Please log into your account, locate the manuscript record, and check for the action link "View Attachments". If this link does not appear, there are no attachment files.]

To ensure your figures meet our technical requirements, please review our figure guidelines: https://journals.plos.org/plosone/s/figures

You may also use PLOS’s free figure tool, NAAS, to help you prepare publication quality figures: https://journals.plos.org/plosone/s/figures#loc-tools-for-figure-preparation.

NAAS will assess whether your figures meet our technical requirements by comparing each figure against our figure specifications.

# Author comment: Author response to Decision Letter 1

**DOI:** 10.1371/journal.pone.0361767.r002

18 Sep 2026

Response to the Academic Editor and Reviewers

Manuscript ID: PONE-D-26-28600

Dear Academic Editor and Reviewers,

Thank you for the careful and constructive review of our manuscript. We revised the paper and its supplementary files point by point. Our responses below explain what was changed and where the revised materials provide the requested evidence.

1. Editorial requirements: title page, ethics, consent, and data availability

Response: A complete title page is included. Methods now identifies the Ethics Review Committee of Qilu Normal University, approval date January 23, 2025, and Ethical Approval No. xsllsc2025-028. Adults clicked “Agree” to provide electronic written consent; a parent or legal guardian used the same recorded procedure for participants younger than 18. S1 Data, S2 File, and S3 Statistical Supplement are identified in the Data Availability Statement.

2. Reviewer 1: clarify the focal “brand”

Response: The focal referent is now explicitly defined as the pianist/master teacher in one specific recalled masterclass. The manuscript also acknowledges that some respondents may have incorporated the series or host institution.

3. Reviewer 1: citation audit

Response: Clearly mismatched or decorative citations were removed or replaced. The five-dimensional relationship-interaction scale is attributed to Zhong and Zhang (2015), and unsupported citations at H1, H2, and H6 were removed. The reference list itself was otherwise preserved and supplemented with directly relevant sources.

4. Reviewer 1: add masterclass scholarship

Response: The Introduction now discusses public masterclasses, observational learning, and the differing learning positions of performers and observers, citing Haddon (2014) and Hanken (2015).

5. Reviewer 1: performer versus observer

Response: Because the questionnaire did not record role, the manuscript explicitly identifies this as a limitation and does not retrospectively classify participants.

6. Reviewer 1: sample framing and income

Response: We removed the adult-only label from the title because the sample included both adults and adolescents. The abstract now describes 382 adult and adolescent participants, and the Methods reports the complete age, education, gender, and music-background distributions. We also added a limitation concerning the predominantly adult, highly educated, music-trained sample. References to income were removed because income variables are not present in the retained analytic dataset.

7. Reviewer 1 and Reviewer 2: measurement and translation

Response: Methods specifies the five Likert response points, construct-level sources, and contextual adaptation. S2 File provides all 26 administered Chinese items and faithful English translations. We transparently state that retained records do not document independent back-translation, expert-panel review, or pilot testing.

8. Reviewer 1: common-method bias

Response: S3 Table 2 reports full-collinearity VIFs for all eight constructs (1.222–1.734), all below 3.3.

9. Reviewer 1: PLS-SEM reporting completeness

Response: Methods now reports 5,000 bootstrap resamples, two-tailed tests, 95% BCa intervals, HTMT, Fornell–Larcker, SRMR = 0.047, and inverse-square-root sample-size reasoning. Table 3 retains every original number; its caption and note now distinguish predictor-specific f² from endogenous-construct R² and Q². Complete additional diagnostics are in S3.

10. Reviewer 1 and Reviewer 2: specific indirect effects

Response: We estimated all ten specific indirect associations with 5,000 two-tailed BCa bootstrap samples; seven confidence intervals excluded zero. S3 Table 3 reports every estimate and interval. Throughout the abstract, hypotheses, results, and discussion, we use “specific indirect association” or “intervening statistical pathway.” We no longer describe music receptivity as a demonstrated mediator, because the cross-sectional design cannot establish temporal mediation.

11. Reviewer 1: fsQCA threshold, anchors, PRI, core/peripheral logic, and robustness

Response: The main manuscript reports all empirical calibration anchors, the PRI criterion, and the parsimonious/intermediate nesting rule. To preserve the validated original result tables, we retained Tables 5–6 in their original form. S3 Table 5 transparently compares that baseline with frequency thresholds of 3 and 5, consistency .82, PRI .65, and alternative calibration. The stricter solutions do not reproduce all seven baseline configurations; we now discuss this sensitivity explicitly.

12. Reviewer 1: configuration-description mismatch

Response: We corrected the Results and Discussion so that M1 is described as the complete configuration displayed in Table 6, explicitly acknowledging courtesy and affective receptivity. We removed the earlier selective description that reduced M1 to relationship energy, brand personality, relationship status, and attentional receptivity.

13. Reviewer 1 and Reviewer 2: causal language

Response: We searched the full manuscript for “influence,” “affect,” “effect,” “lead to,” “enhance,” “cause,” and “causal.” Statements about our findings in the hypotheses, Methods, Results, fsQCA section, and Discussion now use noncausal language such as “associated with,” “positively related to,” “specific indirect association,” and “configuration associated with.” The limitations explicitly address the cross-sectional design, retrospective recall, convenience sampling, and possible reverse or reciprocal relationships.

14. Reviewer 1: PLOS-specific issues

Response: The ethics relationship is explained: Qilu Normal University provided local oversight because recruitment and data collection occurred in China, whereas the Moscow affiliations are academic appointments. Corresponding-author location must be set manually in Editorial Manager to Saint Petersburg, Russia.

15. Reviewer 1: formatting

Response: We corrected the duplicate table numbering. The tables are now numbered consecutively from Table 1 through Table 6, and the reference-list heading is now “References.” We also standardized spacing, Q² notation, caption wording, et al. usage, and American English. Tables remain in a plain black-and-white format.

Files included with the revision

Clean revised manuscript

Highlighted revised manuscript

S1 Data: anonymized analytic dataset

S2 File: complete bilingual questionnaire

S3 Statistical Supplement: HTMT, VIF, SRMR, specific indirect associations, calibration anchors, and fsQCA robustness checks

Ethics approval certification

We appreciate the reviewers’ guidance. The revision has helped us clarify the study’s scope, report the supplementary analyses more transparently, and align the interpretation with what the cross-sectional data can support.

Sincerely,

Zhao Yixuan, Yan Su, and Zhu Xiaohui

Attachment Submitted filename: Response_to_Reviewers_revised.docx

# Peer review: Decision Letter 1

**DOI:** 10.1371/journal.pone.0361767.r003

**Authors:** Chao Cheng

15 Oct 2026

Relational Learning Cues, Music Receptivity, and Performance Self-Efficacy Among Piano Masterclass Participants: A PLS-SEM and fsQCA Study

PONE-D-26-28600R1

Dear Dr. Zhu,

We’re pleased to inform you that your manuscript has been judged scientifically suitable for publication and will be formally accepted for publication once it meets all outstanding technical requirements.

Within one week, you’ll receive an e-mail detailing the required amendments. When these have been addressed, you’ll receive a formal acceptance letter and your manuscript will be scheduled for publication.

An invoice will be generated when your article is formally accepted. Please note, if your institution has a publishing partnership with PLOS and your article meets the relevant criteria, all or part of your publication costs will be covered. Please make sure your user information is up-to-date by logging into Editorial Manager at Editorial Manager® and clicking the ‘Update My Information' link at the top of the page. For questions related to billing, please contact billing support.

If your institution or institutions have a press office, please notify them about your upcoming paper to help maximize its impact. If they’ll be preparing press materials, please inform our press team as soon as possible -- no later than 48 hours after receiving the formal acceptance. Your manuscript will remain under strict press embargo until 2 pm Eastern Time on the date of publication. For more information, please contact onepress@plos.org.

Kind regards,

Chao Cheng

Guest Editor

PLOS One

Additional Editor Comments (optional):

Reviewers' comments:

Reviewer's Responses to Questions

**Comments to the Author**

Reviewer #1: All comments have been addressed

Reviewer #2: All comments have been addressed

**********

2. Is the manuscript technically sound, and do the data support the conclusions?

Reviewer #1: Yes

Reviewer #2: Yes

**********

3. Has the statistical analysis been performed appropriately and rigorously?

Reviewer #1: Yes

Reviewer #2: Yes

**********

4. Have the authors made all data underlying the findings in their manuscript fully available?

The PLOS Data policy

Reviewer #1: Yes

Reviewer #2: Yes

**********

5. Is the manuscript presented in an intelligible fashion and written in standard English?

Reviewer #1: Yes

Reviewer #2: Yes

**********

Reviewer #1: The author has responded to all the issues raised in the previous round of review, and I believe the manuscript can be accepted.

Reviewer #2: The authors have satisfactorily addressed the concerns raised in my previous review. The revised manuscript provides substantially greater conceptual and methodological clarity, particularly with respect to the definition of the focal “brand,” the interpretation and reporting of indirect associations, the use of non-causal language appropriate for the cross-sectional design, and the description of scale adaptation and measurement procedures. The revisions have strengthened the transparency and interpretability of the study, and I have no further substantive concerns. I therefore recommend acceptance.

**********

what does this mean?). If published, this will include your full peer review and any attached files.

If you choose “no”, your identity will remain anonymous but your review may still be made public.

**Do you want your identity to be public for this peer review?**  For information about this choice, including consent withdrawal, please see our Privacy Policy

Reviewer #1: No

Reviewer #2: No

**********

# Editor report: Acceptance letter

**DOI:** 10.1371/journal.pone.0361767.r004

**Authors:** Chao Cheng

PONE-D-26-28600R1

PLOS One

Dear Dr. Zhu,

I'm pleased to inform you that your manuscript has been deemed suitable for publication in PLOS One. Congratulations! Your manuscript is now being handed over to our production team.

At this stage, our production department will prepare your paper for publication. This includes ensuring the following:

* All references, tables, and figures are properly cited

* All relevant supporting information is included in the manuscript submission,

* There are no issues that prevent the paper from being properly typeset

You will receive further instructions from the production team, including instructions on how to review your proof when it is ready. Please keep in mind that we are working through a large volume of accepted articles, so please give us a few days to review your paper and let you know the next and final steps.

Lastly, if your institution or institutions have a press office, please let them know about your upcoming paper now to help maximize its impact. If they'll be preparing press materials, please inform our press team within the next 48 hours. Your manuscript will remain under strict press embargo until 2 pm Eastern Time on the date of publication. For more information, please contact onepress@plos.org.

You will receive an invoice from PLOS for your publication fee after your manuscript has reached the completed accept phase. If you receive an email requesting payment before acceptance or for any other service, this may be a phishing scheme. Learn how to identify phishing emails and protect your accounts at https://explore.plos.org/phishing.

If we can help with anything else, please email us at customercare@plos.org.

Thank you for submitting your work to PLOS One and supporting open access.

Kind regards,

PLOS One Editorial Office Staff

on behalf of

Dr. Chao Cheng

Guest Editor

PLOS One
